# Recent advances in feed and nutrition of beef cattle in China — A review

**DOI:** 10.5713/ab.22.0192

**Published:** 2022-09-06

**Authors:** Qian Gao, Hu Liu, Zuo Wang, Xinyi Lan, Jishan An, Weijun Shen, Fachun Wan

**Affiliations:** 1 College of Animal Science and Technology, Hunan Agricultural University, Changsha 410128, China; 2 State Key Laboratory of Grassland Agro-Ecosystems; College of Ecology, Lanzhou University, Lanzhou, 730000, China

**Keywords:** Beef Cattle, China, Feed Resource Development, Nutrient Requirements, Nutritional Regulation

## Abstract

The beef cattle industry in China has advanced remarkably since its reform and opening up; consequently, China has become the world’s third-largest beef cattle producer. China is also one of the countries with the most substantial research input and output in the field of beef cattle feed and nutrition. The progress and innovation by China in the research field of beef cattle feed and nutrition have undoubtedly promoted the development of the domestic beef cattle industry. This review summarizes recent advances in feed resource development, nutrient requirements, and nutritional regulation of beef cattle in China. Limitations in current research and perspectives on future work are also discussed.

## INTRODUCTION

The vigorous development of beef cattle farming can alleviate the competition for grains between humans and livestock and help improve the dietary structure of humans. Additionally, beef cattle farming contributes to rural revitalization in China. Given these factors, China has been increasing support for beef cattle farming and related research over the past decades.

**Figure 1:**
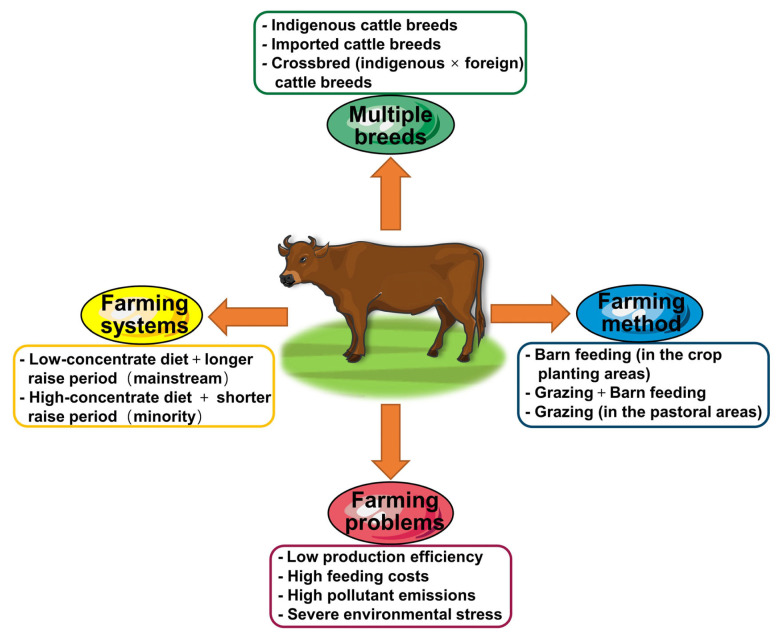
Current status of beef cattle farming in China.

China has become the world’s third-largest beef cattle producer, with an annual beef output of 6.72 million tonnes in 2020 [1]. At present, China’s GDP ranks second in the world, with a per capita GDP of US $12,500. Brazil is the world’s largest beef cattle producer, with a per capita GDP of US $8,000. However, the annual per capita consumption of beef in Brazil is 32.7 kg, more than four times that of China (Statista, https://www.statista.com). The low per capita beef consumption in China is due to the Chinese dietary habit of preferring pork and chicken. Beef cattle industry in China was established late. Before that, cattle, as important draught animals, were forbidden to be slaughtered without permission, which made it difficult to establish beef consumption habits. However, as machines have replaced cattle for ploughing in recent decades, slaughtering cattle for food is no longer restricted. Chinese people have gradually incorporated more beef into their diet, resulting in the sustained and rapid growth of per capita consumption of beef, highlighting China as a promising market. Driven by domestic beef consumption demand, the beef industry in China is expected to further develop and become more prominent in the global beef industry. However, China’s beef cattle industry has been facing multiple issues that restrict its efficient and sustainable development (Figure 1). The production efficiency of the beef cattle industry in China lags behind that of other developed countries, and the total yield of high-grade beef in China is much lower than domestic consumption. This is mainly because of the backward feeding management strategies and cattle breeding. Diets mainly consist of medium- and low-quality roughage, resulting in a low feed conversion rate and slow growth rate of beef cattle. Furthermore, according to the National Breed List of Livestock and Poultry Genetic Resources (2021 Edition), China has 80 beef cattle (*Bos taurus*) breeds, including 55 local breeds, 15 imported breeds, and 10 cultivated breeds. Local beef cattle breeds and their hybrid offspring make up the main body of the beef cattle farming industry in China. However, the body size and production efficiency of most of these breeds are poor. Beef cattle often face various environmental stresses, such as heat and cold stresses, which deteriorate animal welfare and farming profits [2–4]. In addition, beef cattle farming produces many pollutants, particularly methane [5]. In recent years, the Chinese government has promulgated a series of strict environmental protection laws that pose severe challenges to beef cattle farming in China.

Feed and nutrition research plays a vital role in resolving the abovementioned problems, thus promoting the transformation and modernization of the beef cattle industry in China. In this review, we summarize the research progress in this field over the past decade.

## DEVELOPMENT OF FEED RESOURCES

**Figure 2:**
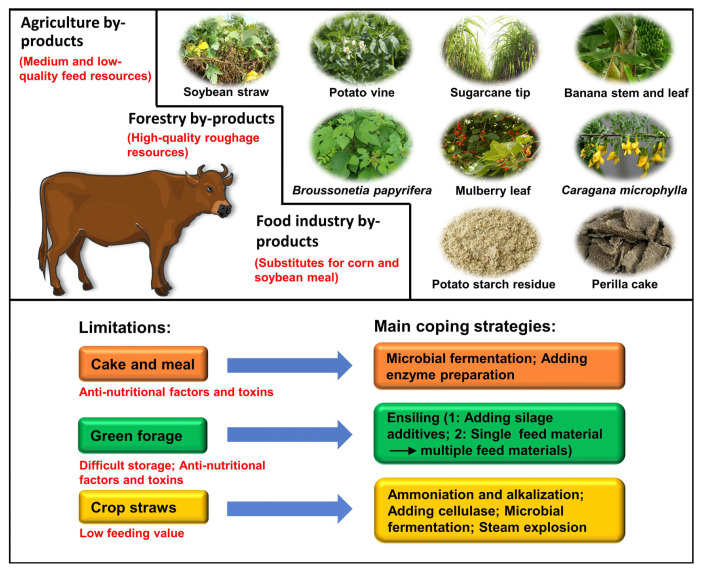
Some unconventional feed resources in beef cattle farming in China, as well as the limitations and coping strategies for the development of unconventional feed resources.

In recent years, the rising costs of raw materials have led to an increase in the costs of feeding beef cattle in China. The agriculture, forestry, and food industry produce many by-products that can be used as cheap feed resources to reduce feeding costs (Figure 2).

Previous studies have reported the nutritional components of these by-products [6,7]. Moreover, numerous experiments have been conducted to evaluate the feeding value of these resources for beef cattle (Table 1). Notably, these resources have not been effectively utilized. The main factors restricting the utilization of these resources in beef cattle farming are their low nutritional value, poor palatability, difficult preservation, and presence of toxic substances and anti-nutritional factors. Therefore, these feed resources are often processed to compensate for their defects. For example, silage treatment is conducive to the long-term preservation of green forage, such as banana stems and leaves [8]. In addition, ammoniation [9] and microbial inoculation [10] can improve the feeding value of crops straw, thus expanding the application of these resources in beef cattle production.

## NUTRIENT REQUIREMENTS OF BEEF CATTLE

Since the promulgation of the “Feeding Standard of Beef Cattle” (FSBC) in 2004, China has continued to strengthen its research on the nutrient requirements of beef cattle, mainly focusing on energy and protein requirements of growing-finishing beef cattle (Table 2). However, marginal information is available on the demand of growing-finishing beef cattle for other nutrients, such as minerals and vitamins. Additionally, Xu et al [11] showed that a phosphorus concentration of 0.37% in the diet can satisfy the phosphorus requirements of crossbred replacement beef heifers (Simmental×Chinese yellow cattle) with body weights of (369.5±37.8) kg.

We compared the conclusions of previous studies with the recommendations of the FSBC [12], and the results suggested that these recommendations may over- or underestimate the actual nutrient requirements for beef cattle (Table 3). For example, Zhang et al [13] reported that the crude protein requirements of Qinchuan cattle with body weights of (289.3±17.8) kg (with an average daily gain of 0 to 1.2 kg) were lower (29 to 132 g per cattle per day) than those recommended by the FSBC [12]. The formulation of FSBC is based on the research results before 2004. Since 2004, China has been vigorously promoting the improvement of beef cattle breeds. During this period, many changes have been made to the composition of beef cattle breeds. For example, China cultivated some new beef cattle breeds after 2004, such as Xianan cattle, Yanhuang cattle and Yunling cattle. In addition, the proportion of concentrate in beef cattle diets is gradually increased to improve their production efficiency. Therefore, the difference between FSBC and the research results may be due to the changes in breeding, feed composition, and feeding management, which can affect the nutritional requirements of beef cattle.

## NUTRITIONAL REGULATION OF BEEF CATTLE

### Nutritional regulation strategies to improve the growth and rumen fermentation of beef cattle

The average production efficiency of beef cattle in China has been lower than that in developed countries for a long time, which reduces the profit and market competitiveness of the domestic beef cattle industry. Improving the production efficiency of beef cattle is thus a critical problem for the beef cattle industry in China.

The growth performance of beef cattle can be improved by adjusting the feed composition in the diet and adding functional substances. For adjusting diet structure, it has been reported that the growth performance and meat quality improved when beef cattle consumed high-quality roughage [14]. Moreover, increasing the dietary energy [15,16] and crude protein [17] levels can help improve the growth performance of beef cattle. Improved dietary nutrient levels may improve the growth performance of beef cattle, but can also cause adverse effects on rumen function, the viscera, and body metabolism of beef cattle [18]. Functional additives, such as probiotics and plant extracts, can improve the growth performance of beef cattle without exhibiting these negative effects (Table 4).

In addition, the rumen, the main digestive organ of ruminants, provides 70% to 80% of the energy requirements for growth. Previous studies have reported that regulating dietary energy [19] and crude protein [20] levels and roughage combinations [21] can affect the rumen fermentation of beef cattle. Diets supplemented with 2-methylbutyrate [22], and folic acid [23] can promote rumen nutrient degradation and mycoprotein synthesis in beef cattle. Moreover, the addition of live yeast [24] and inulin [25] in the diet can increase the ratio of propionic acid to acetic acid in the rumen fluid, thus improving the energy utilization of feed in beef cattle. Additionally, beef cattle are usually fed a high-concentrate diet during the fattening period, which can easily induce subacute ruminal acidosis (SRAS). Dietary supplementation with inulin [25], and niacin [26] can effectively relieve SRAS and improve the rumen fermentation of beef cattle.

### Nutritional regulation strategies to improve the meat quality of beef cattle

Beef quality is one of the most important factors affecting price and consumers’ purchase behavior. In China, the emphasis on the nutritional regulation of beef quality is for improving the intramuscular fat (IMF) content and optimization of meat fatty acid composition. Previous studies reported that increased dietary energy levels can increase the transcription and translation of adipogenic genes, promote the differentiation of preadipocytes into adipocytes, and decrease transcription and translation of lipidolytic genes, thus promoting the accumulation of IMF in beef [27,28]. In addition, dietary supplementation with daidzein [29], nicotinic acid [30], and conjugated linoleic acid [31] can also promote IMF deposition of beef cattle.

Unsaturated fatty acids (UFA), which as an important component of meat, are important for human health [32]. Wang et al [33] showed that increasing dietary energy levels can increase the monounsaturated fatty acids content of beef, which is attributed to a change in the rumen microflora. Furthermore, dietary supplementation with rumen-protected unsaturated fat [34], microalgae [35], and oregano essential oil [36] can increase the UFA content and optimize the fatty acid composition of beef.

### Nutritional regulation strategies to improve the welfare of beef cattle

Heat, cold, and transportation are the most common and harmful sources of stress for beef cattle. Heat stress can impair the antioxidant, immune, and digestive functions of beef cattle [4,37]. Previous studies have reported that optimizing the dietary cation-anion balance [38] and increasing dietary nutrient levels [39,40] can effectively alleviate heat stress in beef cattle. Moreover, Zhuang et al [41] showed that replacing 30% of the forage component with fermented herbal tea residue in the diet can increase fecal microbial diversity and alleviate heat stress. In addition to adjusting the diet structure, diets supplemented with honeysuckle extract [42], grape seed extract [43], creatine pyruvate [44], puerarin [45], rumen-protected γ-aminobutyric acid [46], and *Agastache rugosa* essential oil [47] can alleviate heat stress in beef cattle.

Cold stress can damage the immune and antioxidant functions of beef cattle by activating the hypothalamus–pituitary–thyroid axis [2]. A recent study showed that increasing the dietary energy levels can relieve cold stress in beef cattle [48].

Transport stress can lead to an imbalance in rumen flora, nutritional metabolism, and immunity of beef cattle [3,49]. It has been reported that dietary supplementation with *Astragalus polysaccharides* [50] and creatine pyruvate [51] can alleviate transport stress in beef cattle.

### Nutritional regulation strategies for reduction of pollutant emissions from beef cattle farming

High pollutant emission is a basic characteristic of the current beef cattle farming industry in China. Stricter environmental protection constraints in recent years have thus resulted in severe challenges for the beef cattle industry in China.

Methane (CH_4_) is one of the main pollutants released from beef cattle farming. In 2009, methane emissions from beef cattle farming in China accounted for nearly two-thirds of all CH_4_ produced by domestic ruminant farming [5]. In addition, CH_4_ emissions from ruminants account for 2% to 12% of total dietary energy [52]. Therefore, mitigating CH_4_ emissions is particularly important for the efficient and sustainable development of the beef cattle industry. The nutritional regulation strategies for mitigating CH_4_ emissions can be mainly divided into optimizing the diet structure and adding functional substances to the diet (Table 5).

In addition to CH_4_, beef cattle farming also produces other pollutants such as nitrogen, organic matter, and nitrous oxide (N_2_O). A recent study reported that balancing the dietary ratio of nitrogen to sulfur can help increase nitrogen retention and reduce urinary nitrogen content of beef cattle [53]. Dietary supplementation with rumen-protected methionine [54], vitamin E [55], and N-carbamylglutamate [56] can also increase nitrogen retention and reduce urinary nitrogen content of beef cattle. Although the addition of tannic acid [57] and gallic acid [58] to the diets of beef cattle cannot affect the nitrogen balance, but they can attenuate the urine N_2_O-N emissions by transferring nitrogen from the urine to the feces. Additionally, Gao et al [59] showed that adding red cabbage extract to the diet reduced urine N_2_O-N emissions of beef cattle by increasing urinary hippuric acid excretion.

## CURRENT UNDERSTANDING AND FUTURE PERSPECTIVES

China has abundant unconventional feed resources; however, these resources are not utilized effectively. Therefore, it is necessary to further evaluate the nutritional and feeding values of these resources and study appropriate processing and storage technologies to expand their application in beef cattle farming.

Because of the improvements in intensive beef cattle farming in China, the original extensive feeding management strategy has been gradually replaced with precision feeding techniques. Understanding the precise nutrient requirements of beef cattle is the premise of precision feeding. We compared the conclusions of previous studies with the recommendations of the FSBC [12]. The results suggested that these recommendations may over- or underestimate the actual nutrient requirements for indigenous beef cattle in China. Therefore, the nutrient requirements of indigenous beef cattle should be investigated further to establish precision farming techniques that can prevent potential growth deficiencies and minimize pollutant emissions from beef cattle.

Nutritional regulation plays an essential role in beef cattle farming. Functional additives, especially plant extracts and probiotics, have been the focus of research in China in recent years. In addition, based on the importance of the IMF deposition of beef, numerous studies have been conducted on the relationship between nutrition intervention and IMF deposition [27,29]. However, these studies mainly focused on the effects of nutrition intervention on the apparent meat quality traits, while there were relatively few studies on the regulatory mechanism of IMF deposition. It is necessary to explore the biological and nutritional regulation mechanisms of IMF deposition in the future. In addition, carbon peak and carbon neutrality have become popular topics in political and economic activities in China. Therefore, achieving low-carbon beef cattle farming through nutritional intervention is a promising research area in the future.

## CONCLUSION

In general, China has a relatively solid foundation of research in the field of beef cattle feed and nutrition that strongly supports the development of domestic beef cattle industry. However, there are still many limitations in current research, which restrict the efficient and sustainable development of beef cattle industry. In the future, more studies should focus on developing unconventional feed resources, studying the regulation mechanisms for efficient feed utilization and high-quality beef production, and establishing technologies of precision nutrition and low-carbon farming for beef cattle.

## Figures and Tables

**Table 1:** Nutrient composition of some unconventional feed materials (DM basis) and their application effects in beef cattle farming

Feed materials	Nutrient levels (%)								Annual yield^1)^, million tonnes	Application effects	References

	DM	CP	EE	NDF	ADF	Ash	Ca	P			
Agriculture by-products											
Soybean straw	92.99	5.83	1.61	69.86	58.16	-	1.46	0.18	31.36	Rumen degradation rates of DM, OM, and CP: soybean straw > corn straw	[6]
Sugarcane top	94.24	6.60	0.81	76.53	39.80	-	0.64	0.14	27.03	The rumen degradation rate of DM: sugarcane top> corn straw	[6]
Banana stem and leaf	89.70	6.15	2.13	47.72	42.42	8.93	-	-	27.63	ADG (No effect; Replacement ratio of corn silage in the diet = 100%)	[8, 60]
Peanut vine	90.00	10.20	1.61	44.99	39.16	-	2.42	0.11	20.51	Rumen degradation rates of DM, CP, and NDF: peanut vine > corn straw	[6]
Potato vine	11.97	16.84	4.97	37.16	-	9.46	1.88	0.20	10.25	ADG, DMI, and F/G (No effect; Replacement ratio of corn silage in the diet = 20%)	[61,62]
Food industry by-products											
Potato starch residue	89.17	7.92	0.64	31.94	15.89	4.64	0.55	0.66	-	ADG, DMI, and F/G (No effect; Replacement ratio of corn in the diet = 4%)	[63]
Perilla cake	89.26	34.50	7.72	38.50	18.79	2.71	0.98	0.89	-	DM and NDF digestibilities ↑; Nitrogen deposition ↑ (Replacement ratio of soybean meal in the diet = 100%)	[64]
Forestry resources											
Mulberry leaf	95.26	22.50	2.20	24.71	21.37	14.01	2.42	5.65	-	DMI and ADG ↑; Serum MDA content ↓ (Propotion in the diet = 30%)	[65,66]
* Broussonetia papyrifera* leaf	23.62	17.93	3.25	47.64	30.69	6.39	1.06	0.20	-	ADG ↑; F/G ↓; MCP content ↑; PUFA content of beef meat ↑ (Replacement ratio of corn silage in the diet = 66.17%)	[7]
* * *Caragana korshinskii*	95.08	16.43	3.96	63.03	42.09	5.85	1.25	0.27	-	ADG (No effect); Serum urea nitrogen and alkaline phosphatase content ↓; (Propotion in the roughage = 70%)	[67,68]

DM, dry matter; CP, crude protein; EE, ether extract; NDF, neutral detergent fiber; ADF, acid detergent fiber; Ca, calcium; P, phosphorus; ADG, average daily gain; DMI, dry matter intake; F/G, feed to gain ratio; OM, organic matter; MDA, malonaldehyde; MCP, mycoprotein; PUFA, polyunsaturated fatty acids; ↑, increased; ↓, decreased.

1) Annual yield of by-products = annual yield of agricultural products × by-product coefficient (by-products produced per unit of output of agricultural products). The data for the annual output of agricultural products are from the China Rural Statistical Yearbook 2021 [69] and the China Statistical Yearbook 2021 [1]. The by-product coefficient is determined by referring to Zhu et al [70].

**Table 2:** Nutrient requirements of growing-finishing beef cattle in China

Breeds	Energy requirements^1)^ (MJ/d)	Protein requirements^2)^ (g/d)	References
200 to 225 kg			
Jinjiang cattle	rDE = 35.535×ΔW+6.989	-	[71]
	rME = 33.194×ΔW+2.849		
225 to 250 kg			
Jinjiang cattle	-	rCP = 528.79×ΔW+246.65	[71]
		rDCP = 453.86×ΔW+211.70	
275 to 300 kg			
Xianan cattle	rDE = 0.517×BW^0.75^+0.170×ΔW	rCP = 5.40×BW^0.75^+359.35×ΔW	[72]
	rME = 0.402×BW^0.75^ +36.020×ΔW	rDCP = 2.79×BW^0.75^+260.69×ΔW	
Xiangzhong black cattle	rDE = 0.648×BW^0.75^+33.120×ΔW	rCP = 5.29×BW^0.75^+430.69 ×ΔW	[72]
	rME = 0.506×BW^0.75^+32.150×ΔW	rDCP = 2.66×BW^0.75^+377.95 ×ΔW	
Qinchuan cattle	-	rCP = 5.94×BW^0.75^+470.84 ×ΔW	[13]
		rIDCP = 3.71×BW^0.75^+285.22×ΔW	
Simmental × Guizhou local hybrid cattle	rNE_mf_ = (0.300×ΔW+0.322) ×BW^0.75^	rCP = (6.40×ΔW+6.26)×BW^0.75^	[73]
300 to 325 kg			
Wandong cattle	rME_m_ = 0.522×BW^0.75^	rMP_m_ = 3.93×BW^0.75^	[74,75]
	rNE = (0.348+.291×ΔW)×BW^0.75^	rNP_m_ = 2.63×BW^0.75^	
Jinjiang cattle	rDE = 0.638×BW^0.75^+36.837×ΔW	-	[76]
325 to 350 kg			
Qinchuan cattle	rDE = 0.778×BW^0.75^+37.050×ΔW	-	[77]
	rME = 0.668×BW^0.75^+33.490×ΔW		
350 to 375 kg			
Jinjiang cattle	rDE = 0.770×BW^0.75^+40.088×ΔW		
	rME = 0.645×BW^0.75^+38.603×ΔW	-	[78]
400 to 425 kg			
Xianan cattle	rDE = 0.854×BW^0.75^+16.921×ΔW	-	[79]
	rME = 0.709×BW^0.75^+14.043×ΔW		

1) rDE, requirements of digestible energy; ΔW, daily weight gain in kilograms; rME, requirements of metabolizable energy; rME_m_, metabolizable energy requirements for maintenance; BW, body weight in kilograms; rNE, requirements of net energy; rNE_mf_, requirements of combined net energy.

2) rMP_m_, metabolizable protein requirements for maintenance; rNP_m_, net protein requirements for maintenance; rCP, requirements of crude protein; rDCP, requirements of digestible crude protein; rIDCP, requirements of intestine digestible crude protein.

**Table 3:** Comparison of the conclusions of previous studies with the recommendations of the feeding standard of beef cattle

ADG (kg)	BW (kg)	rCP (g/d)		BW (kg)	rNE_mf_ (MJ/d)		rCP (g/d)					BW (kg)	rNE (MJ/d)	
			
		FSBC^1)^	JJ^2)^		FSBC^1)^	SG^3)^	FSBC^1)^	AX^4)^	XN^5)^	SG^3)^	QC^6)^		FSBC^1)^	WD^7)^
0	225 to 250	320	247	275 to 300	19.37	21.74	372	357	365	423	401	300 to 325	23.21	25.09
0.3		452	405		24.77	27.82	501	486	472	552	542		25.69	31.38
0.6		576	564		28.79	33.90	619	616	580	682	684		28.72	37.67
0.9		691	723		34.18	39.98	731	745	688	812	825		32.50	43.96
1.2		796	881		42.51	46.06	834	874	796	941	966		37.33	50.26

ADG, daily weight gain; BW, body weight; rCP, requirements of crude protein; rNEmf, requirements of combined net energy; rNE, requirements of net energy; FSBC, feeding standard of beef cattle; JJ, Jinjiang cattle; SG, Simmental×Guizhou local hybrid cattle; AX, Angus×Xiangxi crossbred cattle; XN, Xinan cattle; QC, Qinchuan cattle; WD, Wandong cattle.

References:

1) [12];

2) [71];

3) [73];

4) [72];

5) [72];

6) [13];

7) [74].

**Table 4:** Effects of different additives on the growth of beef cattle in China

Additives	Dosage^1)^	Responses^2)^	References
*Saccharomyces cerevisiae*	5 g per cattle per day (viable yeast ≥2×10^10^ CFU/g)	ADG ↑; Rumen MCP and propionic acid contents ↑; CP and NDF digestibilities ↑; Serum IgA and IgM contents ↑	[80]
Yeast culture	150 g per cattle per day	ADG ↑; F/G ↓; Back fat thickness ↑	[81]
*Bacillus amyloliquefaciens C1*	4×10^10^ CFU per cattle per day	ADG ↑; FCR ↑; Serum GH and IGF-1 contents ↑	[82]
Daidzein	400 mg/kg DM	ADG ↑; CP digestibility ↑; Serum GH and IGF-1 contents ↑	[83]
Soybean lecithin	20 g/kg DM	ADG ↑; F/G ↓; Rumen total VFA concentration ↑	[84]
Oregano essential oil	10 g per cattle per day	ADG ↑; The slaughter weight ↑	[85]
Betaine	0.6 g/kg DM	ADG ↑; FCR ↑; Rumen total VFA and NH_3_-N contents ↑	[86]
Niacin	640 mg/kg DM	ADG ↑; F/G ↓; OM, DM, CP, NDF, and ADF digestibilities ↑	[87]
N-carbamylglutamate	40 mg/kg body weight	ADG ↑; FCR ↑	[56]
Pantothenate	0.48 g/kg DM	DMI ↑, ADG ↑; OM, DM, CP, NDF, and ADF digestibilities ↑	[88]
Coated folic acid	4 mg/kg DM	ADG ↑; OM, DM, CP, NDF, and ADF digestibilities ↑	[89]
Lysophospholipid	0.05% DM	ADG ↑; EE and CP digestibilities ↑	[90]
Guanidinoacetic acid	0.48 g/kg DM	ADG ↑; FCR ↑; ADF and NDF digestibilities ↑	[86]

CFU, colony-forming units; DM, dry matter; ADG, average daily gain; MCP, mycoprotein; CP, crude protein; NDF, neutral detergent fiber; IgA, immunoglobulin A; IgM, immunoglobulin M; F/G, feed to gain ratio; FCR, feed conversion ratio; GH, growth hormone; IGF-1, insulin-like growth factor 1; DMI, dry matter intake; VFA, volatile fatty acid; OM, organic matter; ADF, acid detergent fiber; EE, ether extract; ↑, increased; ↓, decreased.

**Table 5:** Effects of different nutritional strategies on mitigation of CH_4_ emissions from beef cattle in China

Strategies	Measures^1)^	Responses	References
Dietary structure optimization	Substituting canola or cottonseed meal for soybean meal	CH_4_ yield (L/d): 13.3% (canola meal) or 32.8% (cottonseed meal) ↓	[91]
	Increasing dietary CP level from 8.15% to 10.67%	CH_4_ yield (L/d): 56.0% ↓	[92]
Functional additives supplementation	Cerium (240 mg/kg DM)	CH_4_ yield (L/d): 25.3% ↓	[93]
	2-methylbutyrate (2 g/kg BW)	CH_4_ yield (L/d): 27.07% ↓	[94]
	Tannic acid (13 g/kg DM)	CH_4_ yield (L/d): 14.7% ↓	[95]
	Nitrate (1% DM)	CH_4_ yield (L/d): 28.5% ↓	[96]
	3-nitropropanol (200 mg/kg DM)	CH_4_ yield (L/d): 25.6% ↓	[97]
	Caffeic acid (40 g/kg DM)	CH_4_ yield (mL/g DM) of *in vitro* fermentation at 48 h: 8.1% ↓ (high-forage substrate)	[98]
	Polyphenols from chestnut involucre (0.2% DM)	CH_4_ yield (mL/d) of *in vitro* fermentation: 8.4% ↓	[99]

CP, crude protein; DM, dry matter; BW, body weight; ↓, decreased.
